# Multiple pyogenic granuloma of the penis in a four-year-old child: a case report

**DOI:** 10.4076/1757-1626-2-7831

**Published:** 2009-09-09

**Authors:** Claudio Spinelli, Martina Di Giacomo, Alessia Bertocchini, Barbara Loggini, Raffaele Pingitore

**Affiliations:** 1 Department of Surgery, University of PisaVia Roma 67, 56126 PisaItaly; 2 Department of Pathology, University of PisaVia Roma 67, 56126 PisaItaly

## Abstract

Pyogenic granulomas are common, acquired, benign vascular lesions of the skin and mucous membranes that can develop both spontaneously and traumatically. We present a unique case of a four-year healthy, uncircumcised boy with multiple pyogenic granuloma on the mucous face of the penis foreskin. Although penile multiple pyogenic granulomas have previously been described in adults, there are no reports of similar problems in children. In this patient, the pathogenesis of the lesions is probably trauma related as reported in the anamnesis. Therapeutic options are discussed.

## Introduction

Pyogenic granulomas (PGs), single or multiple, are lesions of the skin and mucous membranes. This condition can involve a variety of organs, but has rarely been reported to affect the prepuce of the penis. Although penile multiple pyogenic granulomas have previously been described in adults [1], there are no reports of similar problems in children. We present a patient who developed multiple, probably trauma related, PGs of the penis.

## Case presentation

**Figure 1.**
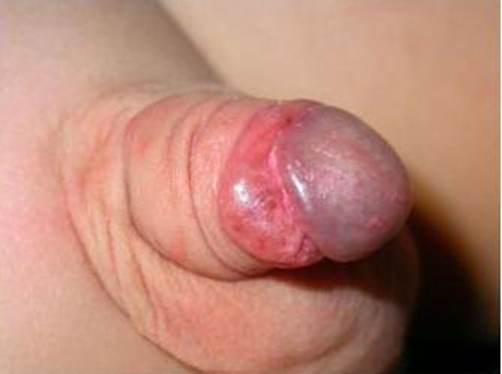
Multiple pyogenic granuloma of the penis, ventral view.

**Figure 2.**
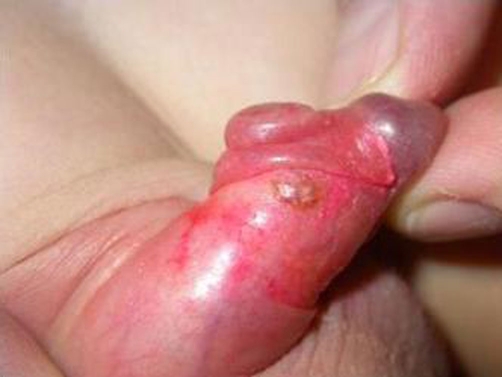
Multiple pyogenic granuloma of the penis, dorsal view.

A previously healthy, 4-year-old, Caucasian-Italian, uncircumcised boy came to our attention for the presence of a little, painless nodular lesion on the mucous face of the dorsal prepuce. The physical examination showed a sessile, bright red - blue, little nodule of around 3 mms in diameter, looking like a very small hemangioma. The history revealed it had appeared around six weeks earlier and it was now enlarging. No others symptoms, either local (bleeding and ulceration) or systemic, were reported and there were no other skin or mucosal abnormalities. Phimosis and Balano Preputial Adhesions (BPA) were absent. It was therefore decided to follow the boy. Twenty days after the first control, the lesion was not just one anymore, but other two satellite nodules occurred. The first lesion appeared bigger than before with a diameter of around 1.2 cm. The new two ones had a diameter of 3 and 5 mm respectively (Figure 1 and Figure 2). After local excision of the lesions, the histopathological examination showed angiomatous tissue composed with congested capillaries and venules which were embedded in an edematous stroma containing a mild chronic inflammatory infiltrate (Pyogenic Granuloma). Even if the pathogenesis of such lesions is not always known, in this patient it is probably trauma related; parents reported traumatic BPA lysis with copious bleeding a few weeks before the appearance of the first lesion. After a follow up of six months no recurrences occurred.

## Conclusion

PGs, also called lobular capillary hemangiomas and teleangiectasic granulomas, are benign vascular proliferations arising from the skin and mucous membranes that may occasionally present intravascularly or subcutaneously. The histopathologic pattern of these lesions is one of capillary lobules separated by fibromyxoid stroma and inflammatory infiltrate, with lymphocytes being the most representative cells.

Even if these lesions can occur in all age groups, commonly PG occurs in children (0.5% of all childhood skin nodules) appearing as bright red - blue, papular or nodular lesions, most often solitary but rarely multiple (principal lesion plus several satellites, erupting after irritation or attempted destruction of the original one - Warner and Wilson - Jones Syndrome) [2]. Most of these growths are small, less than 5 mm in diameter and grow rapidly over several weeks presenting as glistening lesions completely painless but with the tendency to ulcerate and bleed for very little traumas [3].

PG can be classified as an angiogenesis disorder, although the etiology is unknown. Bacterial and viral infections, hormonal stimuli, microscopic arteriovenous anastomoses and angiogenic growth factors are all hypothetical etiological factors [4]. However the latest theories regarding the etiology involve a reactive rather than a neoplastic or infectious process [5]. Traumas, long term irritation and tissue manipulation have recently been proposed as determining and maintenance factors; in a study of 29 PG cases, Michelson [6] showed that half of these were preceded by local injury. Several earlier case reports involving PG of the male genitalia also have involved minor trauma related to zip accident and sexual intercourse [5]. Specialists now postulate an imbalance in angiogenesis regulation as the common pathway for developing of PG [6]. After irritation or trauma, excessive local production of angiogenenic factors or cytokines may incite PG growth [7].

Considering the solitary and most frequent variant, PG, in pediatric age, appears often on the head and neck (62.5% of cases) with frequent involvement of the lips and mucosa, on the trunk (19.7% of cases) and on the extremities, particularly on hands, forearms, palms and soles (17.9%) [7]. Male genitalia location is rare, especially in children. To our knowledge, just two cases of PG affecting the penis in children, have been reported in literature and none of them was about multiple variant. The first case is about a 13 year old phimotic boy who presented a PG on the gland penis. Considering the presence of phimosis and consequent smegma accumulation, the appearance of this lesion has been correlated to chronic irritation [8]. The other case is about a 36 week old boy who developed a PG shortly after circumcision for cultural reasons. It has been supposed to be a complication of wound healing, probably due to excess movement and tension [5]. Conversely, in our case, multiple lesions developed in an uncircumcised, completely healthy boy (without phimosis). Even if the etiology of the lesion in our patients is unknown, parents reported the child underwent a BPA lysis with copious bleeding a few weeks before the appearance of the first lesion. So, we do believe that this traumatic procedure could result in angiogenesis stimulation and PG formation.

Spontaneous involution of PG has been reported, but their removal is often required for aesthetical reasons (especially for facial lesions) and for its easily bleeding and ulceration. There are several treatment options for these kind of lesions; surgical excision followed by linear closer has always been considered the standard one. Other approaches, such as curettage, cautery, cryotherapy and laser, have been recently proposed, but the surgical excision with linear closure seems to be preferable as a consequence of the lower recurrence rate [9]. The possibility to provide samples for histopathological analysis is another advantage. Histopathological confirmation of the diagnosis, in fact, should be mandatory. Even if clinical characteristics combined with a history of a fast growing, easily bleeding lesions are quite often sufficient to distinguish a PG, up to 18% of this kind of lesions are misdiagnosed or confused with other diseases (spiz naevus, true hemangiomas, amelanotic melanoma, Kaposi sarcoma, common warts, squamous cell carcinoma and spindle cell tumors) [9]. A medical therapy, consisting in topical Imiquimod application, has been recently proposed with optimal results; Fallah *et al.* [10] treated 5 children with facial PG using topic Imiquimod 5% cream. In all cases, resolution of the lesion was achieved in 2 14 weeks without recurrences after median follow up 2.3 months.

This pediatric case of multiple PG of the penis prepuce seems to be the first of its kind. The clinical presentation of this boy’s penile lesion is similar to other documented cases of PG in men and may support the role of traumas as an etiology factor underlining the necessity of care in the management of pediatric BPA.

## Abbreviations

BPA: Balano Preputial Adhesions
PG: pyogenic granuloma
